# The potential antiepileptogenic effect of neuronal Cx36 gap junction channel blockage

**DOI:** 10.1515/tnsci-2021-0008

**Published:** 2021-01-22

**Authors:** Guangliang Wang, Xuemei Wu

**Affiliations:** Department of Cardiology, Far Eastern Horizon Hospital, Linghai, Liaoning, People’s Republic of China; Department of Pediatric Neurology, First Hospital of Jilin University, 1 Xinmin Street, Changchun 130000, Jilin, People’s Republic of China

**Keywords:** neuroprotection, Cx36 channel blocker, epilepsy

## Abstract

Epilepsy is one of the most prevalent neurological disorders and can result in neuronal injury and degeneration. Consequently, research into new antiepileptic drugs capable of providing protection against neuronal injury and degeneration is extremely important. Neuronal Cx36 gap junction channels have been found to play an important role in epilepsy; thus, pharmacological interference using Cx36 gap junction channel blockers may be a promising strategy for disrupting the synchronization of neurons during seizure activity and protecting neurons. Based on these promising findings, several *in vivo* and *in vitro* studies are ongoing and the first encouraging results have been published. The results bring hope that neurons can be protected from injury and degeneration in patients with epilepsy, which is currently impossible.

## 1 Introduction

As one of the most common neurobiological disorders, epilepsy is a long-term recurrent disease. Epileptogenesis is a process in which a normal brain transforms into an epileptic brain, generating spontaneous seizures. The term usually refers to the latency period from brain injury or insult to spontaneous seizure or epilepsy [1]. Epileptogenesis is believed to contain three stages: (1) the initial insult or precipitating event, (2) the latent period, and (3) the chronic epilepsy phase [2]. Epilepsy can be associated with neurobiological, cognitive, psychological, and social sequelae, among which neurodegeneration is the most relevant symptom. Many patients with epilepsy experience different degrees of memory or cognitive impairment [3]. Due to the cognitive impairment, behavioral abnormalities, or psychiatric symptoms associated with epilepsy, the daily functioning of patients may be limited. Epilepsy can be controlled using antiepileptic drugs for more than half of the patients. However, most antiepileptic drugs have no neuroprotective effects and epilepsy can easily result in neurodegeneration [4]. Thus, more studies on the pathogenesis and treatment of epilepsy are necessary. The connexin-36 (Cx36) protein, which forms gap junction channels, is the main connexin isoform found in electrical synapses in the brain. Cx36 can influence cytoskeletal microtubule assembly and neuronal cell signaling, playing an important role in the formation of excitatory neural networks and epilepsy. Herein, we focus on the role of Cx36 in epilepsy, especially temporal lobe epilepsy. In particular, we discuss the potential antiepileptogenic effects of neuronal Cx36 gap junction channel blockage, which may be a new approach for epilepsy therapy.

## 2 Gap junctions and connexin

Gap junctions are the most common intercellular connections and play an important role in regulating the growth, differentiation, and proliferation of cells. They are transmembrane passages between adjacent cell membranes that allow some small molecules to pass through (molecules weighing less than 1 kDa or with diameters less than l.5 nm), such as cyclic adenosine monophosphate (cAMP), Ca^2+^, inositol triphosphate (IP3), and adenosine triphosphate [5]. Gap junctions are clusters of intercellular channels, while gap junction channels are composed of two connexons. The junctions are formed by two symmetric connecting bodies, also known as half channels, from the membranes of adjacent cells. Each half channel consists of six connexins [6]. The connexin protein that forms the basic structure of gap junctions is composed of four transmembrane domains (M1–M4), two extracellular loops (E1, E2), and an intracellular loop in which the C-terminal and N-terminus are located [7,8]. The connexin gene encodes a family of proteins. At present, 20 different genes that encode connexin in rodents have been identified, whereas 21 different subunits have been found in humans [9,10]. Gap junction channels enable direct cell-to-cell communication. In the adult and developing central nervous system, there are 12 kinds of gap junction channel proteins with different levels of expression, such as Cx26, Cx32, Cx33, Cx36, Cx37, Cx40, Cx43, Cx45, and Cx46 [11,12]. Cx32, Cx36, and Cx26 are mainly expressed on neurons, while Cx43, Cx30, Cx45, Cx40, and Cx32 are mainly found on astrocytes [13,14,15]. Connexin family members have molecular weights ranging from 26 to 60 kDa with similar structures. Connexins are often named according to the predicted molecular weight. They can be generally divided into three categories: (1) group I or class β connexins are located on liver cells and include Cx26, Cx30, Cx30.3, Cx31, Cx31.1, and Cx32; (2) group II or class α2 connexins are found on myocardial cells and include Cx33, Cx37, Cx40, Cx43, Cx45, Cx46, Cx50, and Cx57; and (3) group III or class γ2 connexins were discovered recently and consist of Cx36 [11].

The opening and closing of gap junction channels can be regulated by a number of factors. The number, distribution, structural changes, and internalization of gap junction channels can all affect the junctions. The behavior of these channels can also be regulated by conformational transformation, aggregation/de-aggregation, and degradation. Protein phosphorylation reportedly contributes to the transmission, assembly, disintegration, degradation, opening, and closing of gap junction channels [15]. The phosphorylation state of gap junction channel proteins can regulate the channels and intracellular phosphatase can catalyze the dephosphorylation of connexin. Additionally, the intracellular Ca^2+^ level can influence the state of gap junction channels. Both a reduction (<10.4 mol/L) and an increase (>10.5 mol/L) in the Ca^2+^ level can reduce the permeability of gap junction channels. The pH of the cytoplasm can also regulate the state of gap junction channels. Additionally, the mechanisms of transcription and regulation are distinctive in different connexins. Even in the same connexin, the mechanisms can be distinctive in different regions. Some of these factors influence the formation of gap junction channels and the number of channels. Chronic exposure of cultured cells to hormones or an imbalance in the hormone levels in the human body can affect the formation and permeability of gap junction channels. Human chorionic gonadotropin, estrogen, and norepinephrine may enhance the connectivity between gap junctions through the elevation of cAMP levels [16]. Hormones can also affect the expression of gap junction channel proteins. Androgens may increase the coupling between neurons in the rat nucleus and affect the formation of electrical synapses in rat spinal motor neurons [17].

## 3 Neuronal Cx36 gap junction channels

Cx36 is a newly discovered member of the connexin family with a molecular weight of about 36 kDa. It was first reported in 1998 by Condorelli et al. and the human Cx36 gene is located on 15ql4 [11,12]. Cx36 is mainly expressed in neurons of the central nervous system, especially interneurons, with almost no expression in astrocytes or oligodendrocytes [11]. Cx36 is highly expressed in the olive nucleus of the brain (inferior olive), particularly in the cornu ammonis (CA)1, CA3, and CA4 subregions of the hippocampus. The Cx36 gap junction channel is most common and serves as the structural basis for coupling between neurons [13]. Thus, Cx36 may play a very important role in electrical signal transformation. Cx36 gap junction channels contribute to the initiation and synchronization of interneuron discharge. Long et al. [14] reported that the Cx36 gap junction channel is very important for the synchronization of neuronal rhythmic activity. Cx36 protein is preferentially expressed in the nervous system and is involved in the process of epilepsy, which has been widely reported in the field of electrophysiology [18].

Direct cell-cell communication mediated by gap junctions contributes to embryonic development as well as to the morphological structure, proliferation, differentiation, and coordination of mature cell clusters. Mutation of the connexin gene is associated with a number of diseases. Cx36 has several functions in neurons: (1) Cx36 is involved in neuronal electrical activity and Cx36-associated gap junction channels, as electrical synapses of neurons facilitate the direct intercellular exchange of ions. Meister et al. [19] reported that the transmission of action potentials between two adjacent ganglion cells is almost zero. The transmission of action potentials occurs directly through the gap junction channels rather than through chemical synaptic vesicles. This direct transmission is attributed to the synchronization of neuronal activity. Electrical contact through the gap junction channels between neurons is an important mechanism for synchronization. (2) Cx36 is involved in neuronal development. Gap junction-mediated coupling between neurons is active in immature nerve cells and neurons shortly after birth. Electrical coupling is common in the developing neural system, even prior to the differentiation of chemical synapses, suggesting that gap junction coupling plays a very important role in the formation of neural networks and the development of the cortex. After the nervous system matures, the number of gap junctions between neurons decreases, but some remain between specific neurons to maintain electrical conductivity, neuronal regeneration, and remodeling [20]. (3) Cx36 participates in the transfer of second messengers such as Ca^2+^, cAMP, and IP3 and some essential metabolites. Second messengers coordinate coupling neuronal activity in the region. Intercellular gap junction channels in the region have high permeability, which allow second messengers to pass through gap junction channels between cells, ensuring the biological cell functions in the region are consistent [10]. (4) Cx36 is involved in neuronal self-identification. Electrical coupling and gap junction plaques can quickly form after the mechanical isolation of two cones from the same cell body. (5) Cx36 is involved in learning and memory. Cx36-knockout mice showed memory impairments that varied according to the complexity of the stimuli presented, suggesting that the neuronal gap junction channel protein Cx36 may be involved in learning and memory processes [21].

## 4 Cx36 gap junction channels in temporal lobe epilepsy

Epilepsy is one of the most prevalent neurological disorders [22]. The temporal lobe is the most epileptogenic region of the brain, and temporal lobe epilepsy is the most common form of epilepsy. The characteristics of temporal lobe epilepsy are as follows [23,24]: (1) The seizures are recurrent and spontaneous. (2) The hippocampus, amygdala, and other components of the limbic system play important roles in the symptoms. (3) There are some pathological changes in the epileptic temporal lobe, such as hippocampus sclerosis. (4) Temporal lobe epilepsy is often resistant to antiepileptic drugs. The etiology and pathogenesis of temporal lobe epilepsy are still not very clear. Substantial evidence has shown that temporal lobe epilepsy begins at the hippocampus and spreads to the entire Papez loop. The hippocampus is an important part of the limbic system, which is not only important in learning, memory, and emotion but also closely related to many neurological and psychiatric disorders. Since the hippocampus, especially the CA1 and CA3 regions, is extremely sensitive to ischemia, anoxia, and excitatory amino acid toxicity, neuronal loss and gliosis are common in the hippocampus. Hippocampal sclerosis was found in up to 70% of epilepsy patients, and complex partial seizures begin from the temporal lobe in 70–80% of cases. Some of these patients can be cured after excision of the hippocampus [25]. Ten patients with intractable epilepsy were treated with electrical stimulation to the hippocampus, resulting in efficient control of complex partial and generalized tonic–clonic seizures for seven of them and a reduction in the number of spikes on the electroencephalogram. The seizures were effectively controlled in three of the ten patients after continued electrical stimulation of the hippocampus, without memory loss [26]. Nine patients with refractory epilepsy were treated with electrical stimulation of the epileptic foci in the hippocampus, i.e., six patients treated with bilateral stimulation and three treated with unilateral stimulation. Seizures were reduced by more than 95% in five patients with normal magnetic resonance imaging results, and by 45–70% in four patients with hippocampal sclerosis after 18 months–7 years of a double-blind follow-up study. None of the patients showed neuronal or psychological side effects [27].

Epilepsy reportedly occurs due to an imbalance between excitatory and inhibitory activities in the central nervous system [28]. Epilepsy is closely related to the abnormal structure and function of ion channels, immune factors, imbalance of neurotransmitters, synaptic transmission, glial cells, and gap junction channels. Abnormal discharge of neurons is the pathological basis for epilepsy [29]. Synchronized electrical activity in the neural network is the basis of neurological transmission and neuronal function, and oversynchronized discharge is the electrophysiological basis of epilepsy. Synchronization in the neuronal network plays an important role in epileptic electrical activities, primarily through gap junctions [30]. The gap junction channels in the central nervous system, which have low impedance, are the basis for intercellular electrical coupling [31].

Fast conduction, low impedance, and short delay times are the features of gap junctions. When a cell is stimulated or receives certain information from the external environment, ions or other regulatory signals are rapidly transferred to neighboring cells through gap junction channels. This is an important synchronization mechanism for electrical synaptic contact in the central nervous system [32,33] and has an important role in the pathogenesis of epilepsy. Connexin enhances gap junction-based electrical conductivity, possibly by increasing the number of electrical synapses, thereby increasing the number of electrical synapses and promoting the synchronous firing of neurons to generate seizures [34]. Epilepsy can occur due to an ultra-synchronized discharge of neurons through gap junctions [35]. As the main connexin in the central nerve system, Cx36 is primarily responsible for synchronization and signal conduction in neurons; and its mutation or malfunction contributes to temporal lobe epilepsy.

## 5 Cx36 channel blockers in temporal lobe epilepsy

Epilepsy can be caused by ultra-synchronized neuronal discharge mediated by gap junction channels. The blockage of these channels can effectively reduce the degree of seizures with potential antiepileptic effects [18,36]. The common gap junction channel blockers are as follows: (1) Nonselective gap junction blockers: intracellular acidification (acid/sodium propionate), long-chain alcohols (heptanol and octanol), anesthesia (fluorine burning and B desflurane), and glycyrrhetinic acid derivatives (carbenoxolone and 18*α*-glycyrrhetinic acid). Carbenoxolone, a common nonselective blocker, is concentration dependent. Medina-Ceja et al. [37] found that seizures can almost be completely prevented 30 min after the injection of carbenoxolone in a 4-aminopyridine (4-AP)-induced model of epilepsy in rats, and the effect can last for up to 120 min. (2) Selective blockers of Cx36 neuronal gap junction channels: quinine, quinidine, and mefloquine. Quinine, an antimalarial drug, was recently identified as a selective blocker of Cx36-associated gap junction channels [38] through a reversible and dose-dependent mechanism. Gajda et al. performed electrophysiological recordings on anesthetized rats and semiquantitative reverse transcriptase-polymerase chain reactions. They found that quinine, as a specific blocker of Cx36-associated gap junction channels, can significantly reduce the expression of Cx36, reduce the discharge frequency and amplitude of epileptic activity in rats, and shorten the duration of seizures [39,40]. Quinine was used for the treatment of epilepsy in children even before it was found to be a gap junction channel blocker [41,42]. Quinine also showed anticonvulsant effects in *in vitro* models of epilepsy developed using rat hippocampal slices exposed to GABA_B_ antagonists, low Ca^2+^, or high K^+^ [43,44]. Gajda et al. reported the antiepileptic effects of quinine in 4-AP-induced models of epilepsy *in vivo* [39], but the corresponding results have rarely been reported *in vivo*. Therefore, research on gap junction channel blockers may become the most anticipated.

Gap junction channel blockage is a potential treatment of epilepsy based on a large number of studies *in vitro* and *in vivo*, although it has not been used as an antiepileptic drug in the clinic [18,36]. Maier et al. found that Cx36-associated electrical synapses play a very important role in certain forms of synchronized activity in the hippocampus (Sharp wave-ripple complexes) as well as in super-synchronized epileptiform discharge [45]. The synchronized rhythmic electrical activity between neurons was significantly reduced after knockout of the Cx36 gene compared to the activity in the control group, suggesting that electrical synapses formed by Cx36 are essential for simultaneous inhibitory activities. The expression level of Cx36 in the model of epilepsy is controversial. Collignon et al. reported that the expression level of Cx36 remained unchanged in patients with intractable epilepsy who received an amygdala hippocampus incision [46]. Another study indicated that the expression level of Cx36 was significantly higher in both the cortex and hippocampus in the kainic acid-induced model of epilepsy [47]. Beheshti et al. reported that the expression level of Cx36 was upregulated in the kainic acid-induced model of epilepsy [48]. Quinine can reduce the amplitude and duration of the discharge 20 min after treatment in a potassium-induced model of epilepsy, but the frequency of spontaneous discharge was higher in the CA1 subregion. Further, the discharge in the CA3 subregion was suppressed 40 min after quinine treatment even though the high-frequency discharge persisted [45,49]. The spontaneous discharge in the CA3 and dentate gyrus subregions can be suppressed by octanol, heptanol, and carbenoxolone *in vitro* in the model of epilepsy [50].

## 6 Conclusion

Gap junction channels play an extremely important role in the formation and development of synchronized neuronal discharge. Targeting gap junction channels, especially the blockage of these channels, is a promising strategy for epilepsy treatment. In contrast to current drug treatments, which showed no neuroprotective effects in clinical trials in recent years, the strategy of using Cx36 gap junction channel blockers has shown some positive effects *in vivo* and *in vitro* studies and appears to be superior and worth investigating. The development of Cx36 gap junction channel blockers is currently the strategy with the greatest promise of success. However, all of the available compounds have limited specificity for connexin channels over other membrane channels or other cellular targets. Therefore, in the future, further screening for more specific gap junction channel blocker compounds is necessary to ensure the blockage of specific connexin subtypes without undesired neurological effects.
